# Metallic Sn spheres and SnO_2_@C core-shells by anaerobic and aerobic catalytic ethanol and CO oxidation reactions over SnO_2_ nanoparticles

**DOI:** 10.1038/srep13448

**Published:** 2015-08-24

**Authors:** Won Joo Kim, Sung Woo Lee, Youngku Sohn

**Affiliations:** 1 School of Chemistry and Biochemistry, Yeungnam University, Gyeongsan 38541, Republic of Korea; 2 Center for Research Facilities & Department of Materials Science and Engineering, Chungnam National University, Daejeon 34134, Republic of Korea

## Abstract

SnO_2_ has been studied intensely for applications to sensors, Li-ion batteries and solar cells. Despite this, comparatively little attention has been paid to the changes in morphology and crystal phase that occur on the metal oxide surface during chemical reactions. This paper reports anaerobic and aerobic ethanol and CO oxidation reactions over SnO_2_ nanoparticles (NPs), as well as the subsequent changes in the nature of the NPs. Uniform SnO_2_@C core-shells (10 nm) were formed by an aerobic ethanol oxidation reaction over SnO_2_ NPs. On the other hand, metallic Sn spheres were produced by an anaerobic ethanol oxidation reaction at 450 °C, which is significantly lower than that (1200 °C) used in industrial Sn production. Anaerobic and aerobic CO oxidation reactions were also examined. The novelty of the methods for the production of metallic Sn and SnO_2_@C core-shells including other anaerobic and aerobic reactions will contribute significantly to Sn and SnO_2_-based applications.

Tin (IV) oxide (SnO_2_) has been studied widely because of its potential applicability to lithium-ion batteries, gas-sensors, solar cells, and catalysts^1^,^2^,^3^. Considerable efforts have been made to control the exposed crystal facets, and synthesize pure and hybridized SnO_2_ materials with a range of morphologies, including hollow nanoparticles, wires, nanorods, nanotubes, nanoparticles, nanosheets, and cubes^4^,^5^,^6^,^7^,^8^,^9^,^10^,^11^,^12^,^13^,^14^,^15^,^16^. C/SnO_2_ hybrid materials were reported to have potential applicability to Li-ion batteries^17^,^18^,^19^,^20^,^21^,^22^. Recently, Liang *et al.* synthesized bowl-like SnO_2_@C hollow nanoparticles to retain the advantages of hollow structures, and showed high performance as an anode material for Li-ion batteries^17^. The SnO_2_ materials reported for applications to Li-ion batteries include hollow SnO_2_^23^, SnO_2_ nanotubes^9^,^24^, nanosheets^12^,^25^, nanoboxes^26^, NiCo_2_O_4_@SnO_2_ hetero-nanostructures^27^, capped Sn/SnO_2_ nanocrystals^28^, sandwich-stacked SnO_2_/Cu nanosheets^29^, graphene/SnO_2_ nanoribbon composites^30^, and SnO_2_@TiO_2_ core-shells^31^, Gas-sensing is an active research area for SnO_2_ nanomaterials^32^,^33^,^34^,^35^,^36^,^37^,^38^,^39^,^40^,^41^,^42^,^43^,^44^,^45^,^46^,^47^. A wide range of materials have been prepared and tested as sensor materials. These include Cu-doped SnO_2_ film for H_2_S sensing^33^, multi-layer SnO_2_ nanoplates^34^ and flower-like SnO_2_ for ethanol sensing^35^, aligned epitaxial SnO_2_ nanowires for ppb-level NO_2_ sensing^36^, p-Te/n-SnO_2_ hierarchical heterostructures^37^ and SnO_2_ hollow spheres for ppm-level CO sensing^38^, hollow SnO_2_ nanofibers^39^ and graphene/SnO_2_ hybrids^40^ for H_2_ sensing, clustered SnO_2_ NPs for toluene detection^41^, and SnO_2_ NP-coated ZnO nanotubes for electrochemical dopamine sensing^42^, For solar cell applications^48^,^49^,^50^,^51^, Dong *et al.* reported that quintuple-shelled SnO_2_ hollow microspheres showed superior light scattering suitable for dye-sensitized solar cells^50^. The (thermal and photo) catalytic activity of SnO_2_ has also been studied actively^52^,^53^,^54^,^55^,^56^,^57^. Several examples include the inactivation of bacteria using fluorinated SnO_2_ hollow nanospheres^52^, rhodamine B treatment using flower-like hollow SnO/Sn_3_O_4_ microspheres^53^, Rhodamine 6G photodegradation using hollow supersymmetric SnO_2_ microspheres^54^. SnO_2_ nanorods with exposed (110) facets were reported to have high CO oxidation activity following a Mars–van Krevelen mechanism, even though the nanorods have a low surface area and a less active surface oxygen species^55^,^56^. Studying the surface reaction on SnO_2_ is extremely important to better understand the sensing and catalytic mechanism and for fabricating the nanostructures. Adsorption is a common first step for both sensing and catalytic reactions. Jeong *et al.* reported that nanotextured SnO_2_ surfaces could be produced using a self-catalytic growth method with different oxygen concentrations and annealing temperatures^58^. Müller *et al.* reported showed that the precursor chemistry was important for controlling the morphology and composition of SnO_2_ nanowires^59^.

The specific aim of this study was to identify the changes in morphology and crystal phase of SnO_2_ NPs after aerobic and anaerobic oxidation reactions. This paper reports a new methodology for the production of metallic Sn spheres and SnO_2_@C core-shells. The core-shell structures have very high potential applicability to gas sensing and Li-ion batteries. Metallic Sn spheres were produced from SnO_2_ by an anaerobic ethanol oxidation reaction below 600 °C, which is a significantly lower temperature than that used in the high temperature (>1000 °C) carbothermal reduction method in industry. In addition, the high CO oxidation activity of SnO_2_ NPs has potential applications to catalysis.

## Methods

SnO_2_ NPs were synthesized using a facial hydrothermal method. Briefly, 10.0 mL of 0.1 M SnCl_4_·5H_2_O and 20 mL of deionized water (18.2 MΩ-cm resistivity) were fully mixed in a Teflon bottle. An appropriate amount of 0.1 M NaOH solution was then added to induce precipitation. The bottle was tightly capped and placed in an oven at 120 °C for 12 hours. The reaction bottle was cooled naturally and the white precipitate was collected by centrifugation. The precipitates were washed several times with deionized water and ethanol, and dried in an ambient oven at 70 °C. The morphology and size of the dried powder samples were examined by transmission electron microscopy (TEM, Hitachi H-7600) operated at 100.0 kV. A high resolution TEM image was obtained using a FEI Tecnai G2 F20 S-TWIN at an applied voltage of 200.0 kV. The surface morphology and the chemical composition were examined by scanning electron microscopy (SEM) and energy dispersive X-ray (EDX) analysis, respectively using a Hitachi S-4100 SEM/EDX. The X-ray diffraction (XRD) patterns for the powder samples were obtained using a PANalytical X’Pert Pro MPD diffractometer using Cu Kα radiation. The crystal structures of the metallic Sn spheres were tested by both powder and single crystal X-ray diffraction experiments. Powder X-ray diffraction was conducted using a Bruker AXS D8 diffractometer with a Cu Kα radiation source (40 kV and 40 mA) using a Linxeye 1-D detector. Single crystal X-ray diffraction was conducted using a Bruker AXS APEX II CCD-single crystal diffractometer with a Mo Kα radiation source (50 kV and 30 mA, point beam) and a CCD detector system. The diffuse reflectance absorption spectra for the powder samples were measured using a double beam Neosys-2000 UV–Vis spectrophotometer (Scinco). X-ray photoelectron spectroscopy (XPS) was performed using a Thermo-VG Scientific MultiLab 2000 with a monochromatic Al *K*α X-ray source (1486.6 eV) equipped with a hemispherical energy analyzer. The photoluminescence spectra were obtained using a SCINCO (Seoul, South Korea) FluoroMate FS-2 spectrometer. Raman spectra were obtained using a Bruker Senterra Raman spectrometer at an excitation laser wavelength of 532 nm. The attenuated total reflection (ATR) Fourier transform infrared (FTIR) spectra were obtained using a Thermo scientific Nicolet iS10 spectrometer. The Brunauer-Emmett-Teller (BET) surface areas of the as-prepared powder samples were measured using a Quantachrome ChemBET TPR/TPD analyzer equipped with a thermal conductivity detector. Fig. S1 in the Supporting Information presents the schematics of the experimental setup for aerobic and anaerobic oxidation reactions. Briefly, for aerobic ethanol oxidation reaction, ethanol vapor was introduced into the catalyst by flowing 5% O_2_/N_2_ gas at a flow rate of 40 mL/min. Pure N_2_ gas was used for the anaerobic ethanol oxidation reaction. The reaction products were examined using a SRS RGA200 quadrupole mass spectrometer. The temperature-programmed aerobic CO oxidation experiments were performed by flowing (40 mL/min) a CO (1%) and O_2_ (2.5%) in N_2_ gas mixture to the catalyst powder sample. For anaerobic CO oxidation, 5% CO/N_2_ gas was flowed instead. The sample (20 mg) was placed in a U-quartz tube (4 mm inner diameter) for each oxidation experiment.

### Results and Discussion

**Figure 1:**
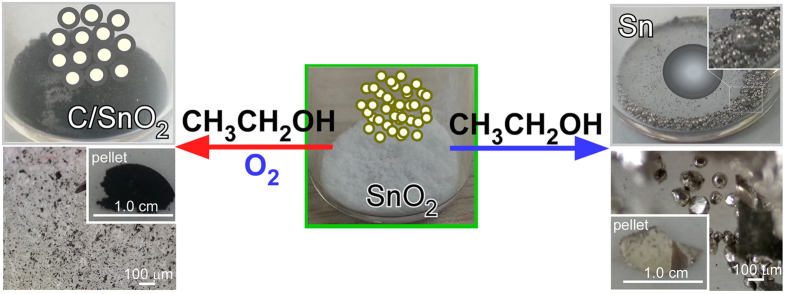
Photograph (top) and optical microscopy (bottom) images for the production of SnO_2_@C core-shells (left, black in color) and metallic Sn spheres (right, silvery-white in color) by aerobic (left) and anaerobic (right) ethanol oxidation reactions over SnO_2_ NPs (middle, white in color), respectively. The insets (left and right bottom two) in the optical microscopy images show photographs of the corresponding pelletized samples.

Figure 1 shows a photograph and optical microscopy images of the samples produced after the ethanol oxidation reaction under aerobic (with O_2_) and anaerobic (without O_2_) conditions. The morphology changed significantly after the reactions. Upon the ethanol oxidation reaction under aerobic conditions, the white powder sample changed to a black powder. Interestingly, under anaerobic conditions, all the power samples changed to silvery-white spheres, indicating complete reduction to Sn metal. Several of the spheres were a few mm in size. To clearly show the metallic Sn spheres, a larger photograph is supplied in the Supporting information, Fig. S2.

**Figure 2:**
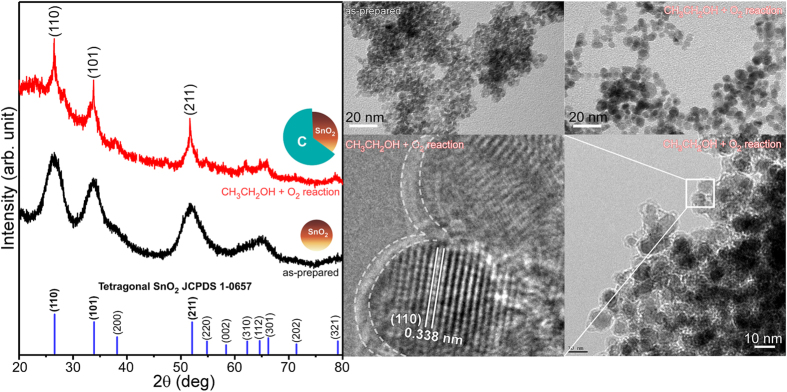
XRD patterns (left) and TEM/HRTEM (right) images of SnO_2_ NPs before and after the aerobic ethanol oxidation reaction. The SnO_2_@C core-shells were formed upon the aerobic reaction.

Figure 2 presents the XRD patterns of the SnO_2_ NPs before and after the aerobic ethanol oxidation reaction. All XRD peaks matched the standard crystal planes of tetragonal SnO_2_ (JCPDS 1–0657), as displayed in Fig. 2. SnO_2_ was likely obtained via Sn^4+^ + OH^−^ → Sn hydroxides → SnO_2_ + H_2_O during the hydrothermal reaction^10^,^15^. The three major XRD peaks at 2θ = 26.5, 33.8 and 51.7° were assigned to the (110), (101), and (211) reflection planes of tetragonal SnO_2_, respectively. The XRD peaks of the as-prepared sample were quite broad, indicating smaller particle sizes. The NP size was estimated using the Scherer formula, d = Kλ/L cos θ, where K is the Scherer constant and L is the full width at half maximum of a reflection. The calculated particle size was in good agreement with the mean size (~2 nm) obtained from the TEM images. Li *et al.* also employed a similar hydrothermal (180 °C for 24 hrs) method to synthesize SnO_2_ with reaction recipes of 1.0 mmol SnCl_4_·5H_2_O, 20 mL deionized water, 10 mL NaOH (1.5 M) solution, and 10 ML ethanol^60^. They obtained nanoparticle-aggregated hierarchical SnO_2_ hollow microspheres (600–900 nm) and understood by dissolution–recrystallization process^60^,^61^ with two major controlling factors of pH and reaction temperature.

For the SnO_2_@C core-shells, the XRD peak became sharper, indicating an increase in crystallinity and/or particle size. The TEM images illustrate the high uniformity and size of the SnO_2_ NPs before and after the aerobic ethanol oxidation reaction. A clear lattice spacing of 3.38 Å was obtained, which is indicative of the single crystal nature. The spacing corresponds to the distance between the adjacent (110) planes of tetragonal SnO_2_^25^,^30^. Alaf *et al.* and Uysal *et al.* employed an alternative method to obtain core-shell type structures, which were tested as anode materials for Li-ion batteries^62^,^63^,^64^. They thermal-evaporated metallic Sn on multiwalled carbon nanotube (MWCNT), and obtained Sn/SnO_2_/MWCNT and Sn/MWCNT nanocomposites with and without plasma oxidation treatment, respectively. The grain sizes of Sn and SnO_2_ were controlled by varying thermal evaporation and plasma oxidation conditions.

The as-prepared SnO_2_ NPs were characterized by UV-Vis absorption spectroscopy, photoluminescence spectroscopy, FT-IR spectroscopy, and BET surface area measurements. A direct band gap of 3.4 eV was measured from the reflectance absorption spectrum and a plot of (αhν)^2^ versus hν (Supporting Info. Fig. S3)^53^. The photoluminescence spectrum was recorded for the as-prepared SnO_2_ NPs at an excitation wavelength of 285 nm (Supporting Info. Fig. S4). Broad emission peaks were observed between 350 and 600 nm, which have commonly been attributed to oxygen defects (or vacancies)^45^,^65^,^66^. The as-prepared SnO_2_ NPs exhibited a large amount of adsorbed water, based on the broad FT-IR peak at 3400 cm^−1^ (Supporting Info. Fig. S5)^38^. The broad peak was almost completely diminished after the aerobic ethanol oxidation reaction at temperatures up to 600 °C. The SnO_2_ NPs (~2 nm) showed a BET surface area of 197.5 m^2^/g, which is significantly larger than the surface area of 38.3 m^2^/g for a reference (>100 nm, Sigma-Aldrich) SnO_2_ powder. For comparison, Xi *et al.*^5^ and Wang *et al.*^25^ reported surface areas of 191.5 m^2^/g for ultra-small (2nm) SnO_2_ nanorods and 180.3 m^2^/g for SnO_2_ nanosheets, respectively.

**Figure 3:**
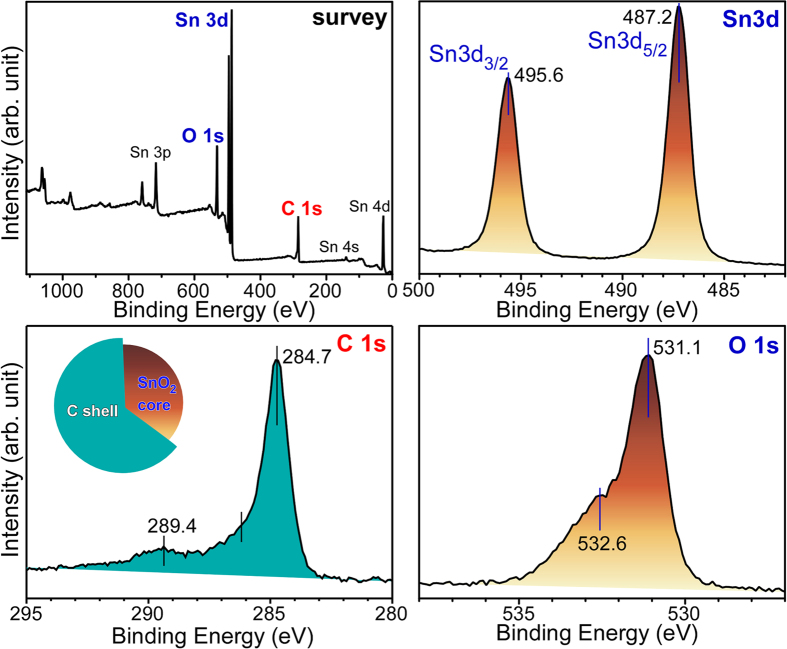
Survey (top left) and high resolution XP spectra of Sn 3d, C 1s and O 1s regions for SnO_2_@C core-shells prepared by an aerobic ethanol oxidation reaction over SnO_2_ NPs.

The X-ray photoelectron spectra (Fig. 3) were recorded to further examine the chemical nature of the SnO_2_@C core-shell nanostructures. The survey spectrum revealed the chemical information of Sn, O and carbon with no other elements. The prominent Sn 3d_3/2_ and Sn 3d_5/2_ XPS peaks were observed at 487.2 ( ± 0.1) eV and 495.6 ( ± 0.1) eV, respectively, with spin-orbit splitting of 8.4 eV, due to the Sn^4+^ oxidation state of SnO_2_^14^,^31^,^55^. These binding energies (BEs) showed no critical difference from those of the as-prepared SnO_2_ nanoparticles. This suggests that the overlayer carbon had been physisorbed on the SiO_2_ surface. A major C 1s XPS peak was observed at 284.7 eV, due to elemental carbon. The two smaller C 1s peaks at 289.4 and 286 eV were assigned to O = C-O and C-OH/C-O-C species, respectively. These chemical species are related to the formation of ethylacetate and aldehyde after the aerobic ethanol oxidation reaction, as further discussed in detail below. Two O 1s XPS peaks were observed at 531.1 and 532.6 eV due to lattice oxygen (O^2−^) of SnO_2_ and adsorbed oxygen (O^−^ abd O_2_^−^) species (e.g., H_2_O and OH), respectively^14^. The adsorbed oxygen species were confirmed by FT-IR spectroscopy (Supporting Information, Fig. S5). The thickness of the carbon overlayer was estimated using a well-known equation^67^, *I* = *I*_0_exp(−d/λ), where d is the shell thickness, λ is the electron inelastic mean-free path (IMFP)^68^, *I* and *I*_*o*_ is the Sn 3d XPS intensities before and after carbon shell formation, respectively. The thickness of the carbon overlayer was calculated to be 1.2 nm using an IMFP (λ) of 2.7 nm^68^.

The SnO_2_@C core-shells were briefly tested as an oxygen sensor material (Supporting Info. Fig. S6) using a two-point probe resistance measurement method^43^. Using a SnO_2_@C pellet, a surface resistance of 6 kΩ was measured at room temperature, which is an extremely low resistance compared to that of bare SnO_2_. The resistance decreased gradually with increasing temperature and was 3 kΩ at 80 °C. The resistance increased upon exposure to oxygen, and showed a good response to the changing oxygen concentration (0.5–5.0%).

**Figure 4:**
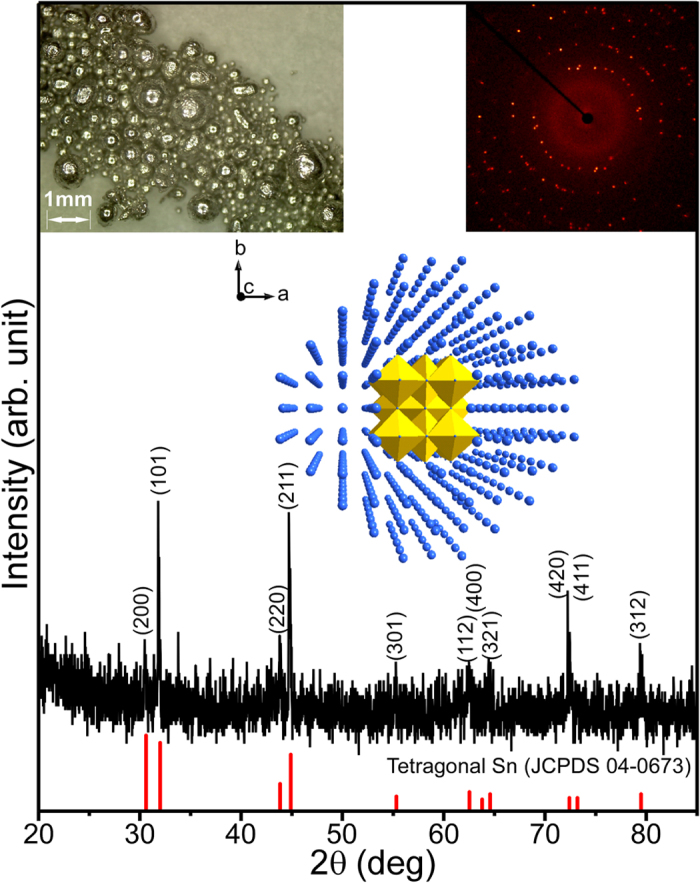
XRD patterns of metallic Sn formed by the anaerobic ethanol oxidation reaction. Optical microscopy image (top left inset), body centered tetragonal Sn crystal structure (middle inset), and crystal pattern of Phi 360 image (top right inset).

The CCD-SCD and PXRD analyses of the Sn spheres produced by the anaerobic ethanol oxidation reaction were obtained in Fig. 4. The very sharp XRD patterns matched those of tetragonal Sn (JCPDS 04-0673)^58^,^62^,^63^,^64^. The diffraction image (inset in Fig. 4) showed single and polycrystalline natures of Sn spheres. Various crystal patterns of Phi 360 degrees obtained for other Sn spheres are provided in the Supporting Information (Fig. S7).

**Figure 5:**
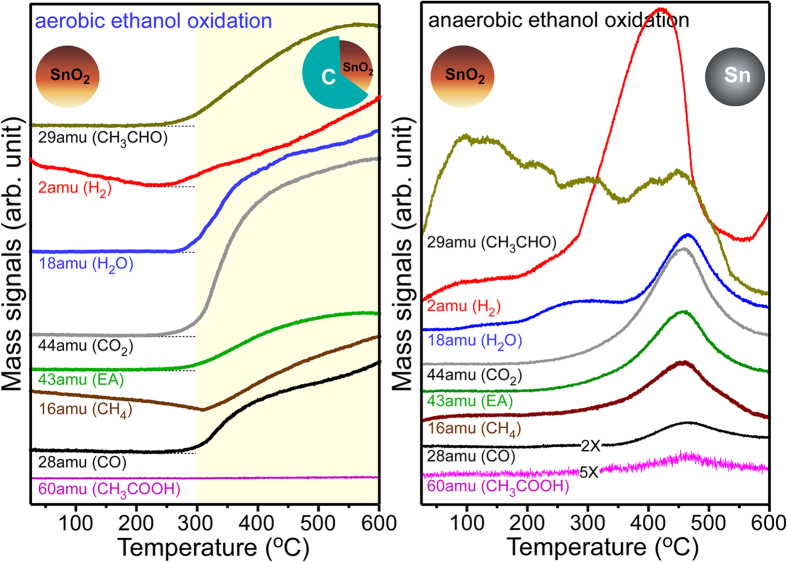
Mass profiles of the chemical species detected during the aerobic (left) and anaerobic (right) ethanol oxidation reactions over the SnO_2_ nanoparticles. SnO_2_@C core-shells were formed under aerobic conditions, whereas metallic Sn spheres were produced under anaerobic conditions.

The reaction products produced during the aerobic and anaerobic ethanol oxidation reactions at different temperatures were examined by temperature-programmed mass spectrometry, as shown in Fig. 5. The quantity of reaction products was not measured, and only the chemical species are discussed. Under aerobic conditions, the mass signals commonly began to increase at ~250 °C, and the intensities kept increasing with in temperature. Based on the mass profiles, the oxidation gas products include hydrogen (H_2_), water (H_2_O), carbon dioxide (CO_2_), carbon monoxide (CO), aldehyde, (CH_3_CHO), methane (CH_4_), acetaldehyde (CH_3_CHO), and ethylacetate (CH_3_COOCH_2_CH_3_, EA) with no acetic acid (CH_3_COOH). On the basis of the literatures^69^,^70^,^71^ and the present results, we propose the following simplified mechanism.


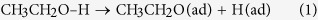



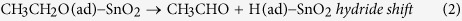



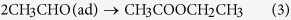



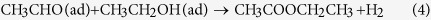



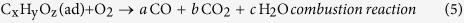



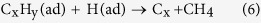


In reaction (1), the O–H bond of ethanol dissociates and adsorbs as H and CH_3_CH_2_O. The adsorbed CH_3_CH_2_O desorbs as CH_3_CHO (acetaldehyde) by a hydride shift reaction in (2). The acetaldehyde in (3) and (4) further proceeds to form CH_3_COOCH_2_CH_3_ (ethylacetate). In reaction (5), CO, CO_2_, and H_2_O are formed by a combustion reaction. Elemental carbon and CH_4_ are formed in reaction (6).

Under anaerobic conditions, although the reaction products were similar, the mass profile curves showed remarkable difference with temperature. Hydrogen and aldehyde were evolved earlier than the other reaction products, possibly due to CH_3_CH_2_O–H → CH_3_CHO + H_2_. At above 450 °C, the mass signals (or the catalytic activity) decreased suddenly and showed the maximum intensities at approximately 450 °C. This suggests that SnO_2_ changes metallic Sn at above 450 °C via the reactions of C_x_H_y_ (ad)−SnO_2_ → *a* CO_2_ + *b* H_2_O + *c* H_2_ + Sn, as discussed above. Because the reaction was performed in the absence of oxygen the oxidation of metallic Sn was less likely. Because the melting point (~230 °C)^58^ of Sn is lower than the reaction temperature of ~450 °C (Fig. 5, right), the reduced Sn may form a spherical droplets of liquid Sn. Upon cooling, the liquid droplet would change to solid Sn spheres, as shown in Fig. 1. A very weak acetic acid signal was also observed under anaerobic conditions, possibly due to the reaction, CH_3_CHO + O_s_ → CH_3_COO(ad) + H(ad) → CH_3_COOH, where O_s_ is the adsorbed oxygen species. The anaerobic ethanol oxidation reaction was also performed with 50× larger SnO_2_ NPs (<100 nm) (Supporting Info. Fig. S8). Although the maximum peaks were observed at a slightly higher temperature of 500 °C, the mass profiles were similar to those of the reactions with 2 nm SnO_2_ NPs. metallic Sn spheres were also obtained after the anaerobic ethanol oxidation reaction with the larger NPs.

In industry, metallic Sn is commonly produced from SnO_2_ via a carbothermal reaction (SnO_2_ + C → Sn + CO_2_) at temperatures above 1200 °C, where carbon is used as the reducing agent. As a green method, a hydrogen reduction [SnO_2_ + 2H_2_ (g) → Sn (s, l) + 2H_2_O (g)] method is used at high hydrogen pressures (>30 kPa) and temperature (>700 °C)^72^. Zhang *et al.* prepared metal Sn nanobelts from SnO_2_ by a substitution reaction using Zn powders in a furnace temperature of 1200 °C^73^. Compared to these two methods, the present anaerobic ethanol oxidation reaction requires a much lower temperature under ambient pressure.

**Figure 6:**
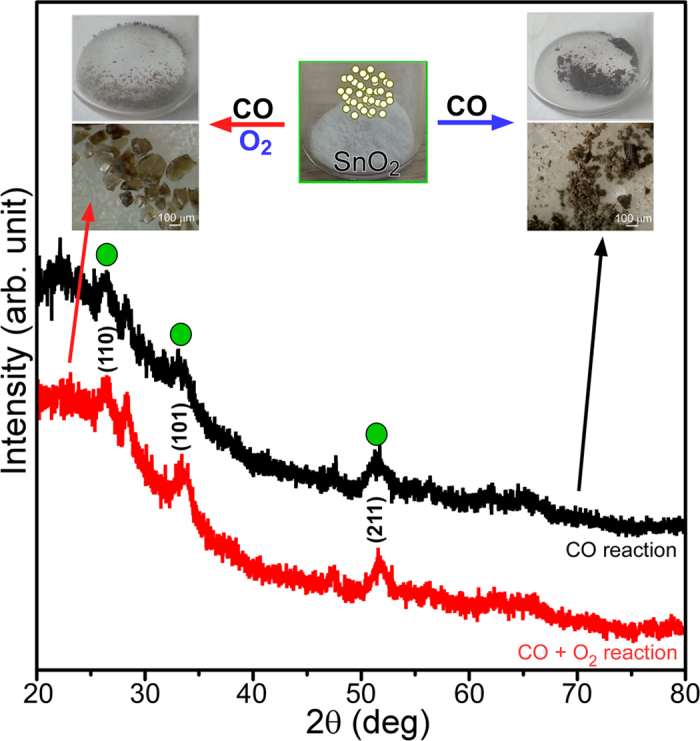
XRD profiles of SnO_2_ after the anaerobic (5% CO in N_2_) and aerobic (1% CO + 2.5% O2 in N_2_) CO oxidation reactions. The inset shows photographs and optical microscopy images of SnO_2_ (brown semitransparent crystals and clusters) by the aerobic (left) and anaerobic (right) CO oxidation reactions. The as-prepared SnO_2_ nanoparticles (white in color) are shown in the middle.

To demonstrate the changes in morphology and crystal phase, another chemical reaction over the NPs was also tested, e.g., aerobic and anaerobic CO oxidation reactions. CO initially adsorbs on the oxide surface, then reacts with surface oxygen and desorbs as CO_2_ (g). The surface oxygen vacancy is replenished by the adsorption of residual (molecular) oxygen; CO (g) + Sn-O_s_ → CO_2_ (g) + Sn-□_vac_ Sn-□_vac_ + 1/2O_2_ (g) → Sn-O_s_, where Sn-□_vac_ = oxygen vacancy and Sn-O_s_ = surface oxygen species^15^. The morphology and/or crystal structure is expected to change differently according to whether there is sufficient replenishment of surface oxygen. Figure 6 shows the XRD patterns and corresponding photographs and optical microscopy images. The color and morphology showed significant changes. Larger brown semitransparent crystals appeared to be formed under aerobic conditions, whereas the aggregated power form was formed under anaerobic conditions. The corresponding SEM images are provided in the Supporting Information Fig. S9. The corresponding XRD peaks became very weak, indicating poor crystallinity, but still showed the reflection planes of tetragonal SnO_2_.

**Figure 7:**
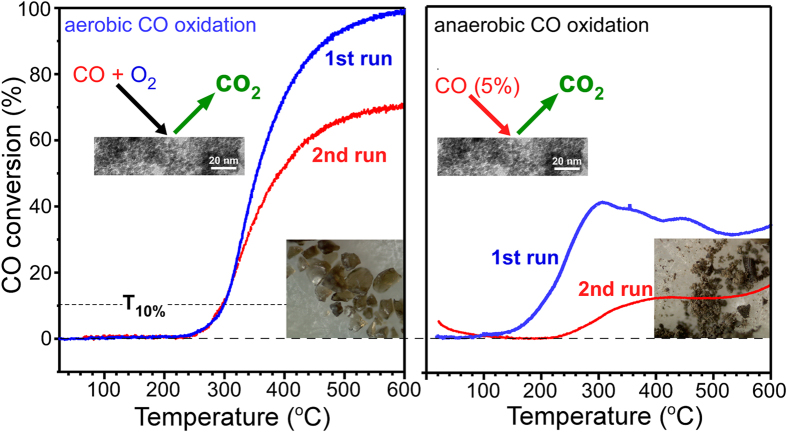
First and second runs temperature programmed CO oxidation conversion (%) profiles of the aerobic (left) and anaerobic (right) CO oxidation reactions over SnO_2_ nanoparticles, where CO conversion (%) = {([CO]_in_-[CO]_out_)/[CO]_in_}×100, T_10%_ = the temperature at 10% CO conversion. CO (1%)/O_2_ (2.5%) in N_2_ and 5% O_2_ in N_2_ were used for the aerobic and anaerobic CO oxidation reactions, respectively.

Figure 7 displays the CO conversion (to CO_2_) profiles of the aerobic and anaerobic CO oxidation reactions. For the first CO oxidation run under aerobic conditions, the oxidation onset temperature and T_10%_ were observed at 230 °C and 300 °C, respectively. This study showed that the SnO_2_ NPs have comparable CO oxidation activity to that reported in the literature^55^. Compared to the first run, the reaction temperatures in the second run were the same between CO conversion of 0% and 20%. Above 20%, however, the conversion differed according to the reaction temperature. This suggests that the CO conversion is critically affected by heat and/or mass transfer limitations at a higher temperatures^74^,^75^. The CO conversion was lower for the second run at higher temperatures. At 600 °C, the catalytic activity was degraded by approximately 30% compared to that of the first run. The degraded activity was attributed to the change in surface area and crystallinity, based on the microscopy images and the XRD patterns. An activation energy (E_a_) of 104.7 kJ/mol was obtained in the CO conversion range of 10–15% from the Arrhenius plot (Supporting Info. Fig. S10). Under anaerobic conditions, CO conversion (%) was much lower than that under aerobic conditions, as expected. The conversion efficiency was more degraded in the second run. Compared to the anaerobic ethanol oxidation, the CO oxidation showed no critical change in crystal phase. This suggests that CO is not a good reducing agent for SnO_2_. A water signal was also detected during the first runs for both the aerobic and anaerobic reactions (Supporting Info. Fig. S11), but not during the second runs. This was expected because the as-prepared SnO_2_ NPs contain water (based on the FT-IR spectrum). For the weaker water signal during the aerobic reaction, it was assumed that SnO_2_ was dehydrated by a stream of oxygen before running the CO oxidation.

The BET surface area was measured to be 81.2 m^2^/g after anaerobic CO oxidation. The surface area was decreased to 70.0 m^2^/g after aerobic CO oxidation reaction. We measured a surface area of 92.5 m^2^/g for SnO_2_@C core-shells formed after aerobic ethanol oxidation reaction. Compared with the surface area of 197. 5 m^2^/g for the as-prepared SnO_2_ NPs, the substantial decrease is due to an increase in particle size. Energy dispersive X-ray (EDX) analysis was performed for SnO_2_ NPs after the anaerobic and aerobic CO and ethanol oxidation reactions (Supporting Info. Fig. S12). For metallic Sn formed after anaerobic ethanol oxidation, the EDX spectrum showed mainly Sn with minor impurity C signal. For SnO_2_@C core-shells formed after aerobic ethanol oxidation, significant C, O and Sn EDX signals were observed as we expected. For the two samples after CO oxidation reactions, the EDX spectra showed similar Sn and O signals, but the sample after anaerobic CO oxidation showed a presence of carbon.

**Figure 8:**
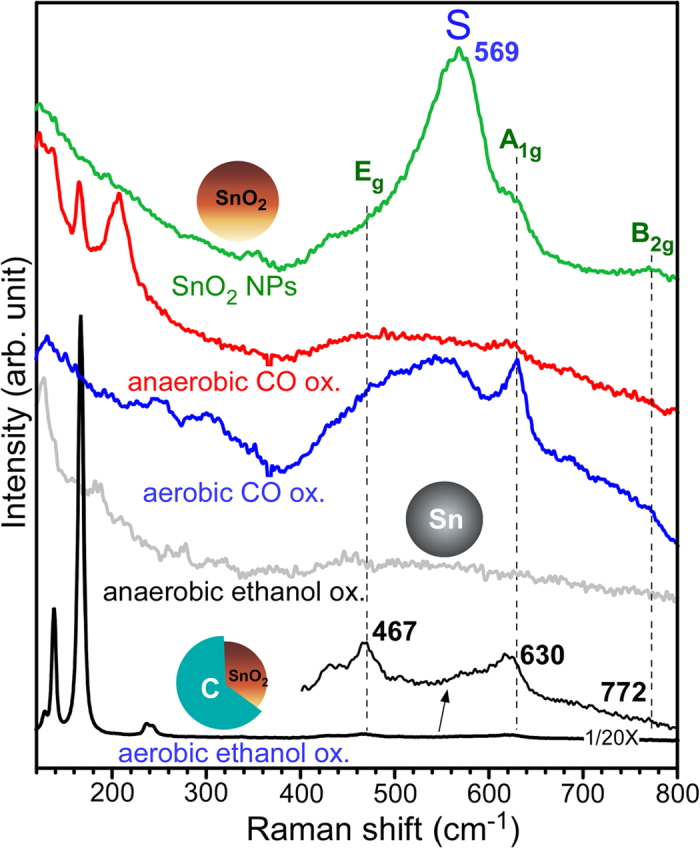
Raman spectra of SnO_2_ NPs before and after the anaerobic and aerobic CO and ethanol oxidation reactions. A laser intensity of 0.25 mW was used for SnO_2_@C core-shells, and 5 mW was used for other samples.

Raman spectroscopy was further employed to examine the structural and chemical states displayed in Fig. 8. For tetragonal SnO_2_ with a space group of *D*_4*h*_, the vibrational modes were ascribed to 1A_1g_ + 1A_2g_ + 1A_2u_ + 1B_1g_ + 1B_2g_ + 2B_1u_ + 1E_g_ + 3E_u_, where A_1g_, B_1g_, B_2g_, and E_g_ are Raman active modes^15^. For the as-prepared SnO_2_ NPs, three peaks were found at 467, 630 and 772 cm^−1^, assigned to E_g_, A_1g_ and B_2g_ vibrational modes, respectively. Interestingly, a broad and strong peak was observed at 569 cm^−1^, which was attributed to surface-related defects^15^,^76^. The peak has commonly been observed and well known to increase with decreasing particle size^4^,^5^,^15^,^44^. Upon anaerobic CO oxidation, the peaks were substantially decreased and showed no clear vibrational modes, indicating formation of very poor crystalline structure. However, after aerobic CO oxidation reaction the A_1g_ peak was more clearly observed as generally expected for SnO_2_ NPs^15^,^44^. Upon anaerobic ethanol oxidation, no Raman signal was observed, in good consistent with the literature for metallic Sn^77^. For SnO_2_@C core-shells formed after aerobic ethanol oxidation, new strong Raman signals was observed, attributed to the shell-carbon species. The smaller peaks at 467 and 630 cm^−1^ were attributed to the E_g_ and A_1g_ vibrational modes of the core SnO_2_, respectively.

## Conclusion

Metallic Sn spheres can be produced by a simple anaerobic ethanol oxidation reaction over SnO_2_ (2 nm size, BET surface area = 197.5 m^2^/g, band gap = 3.4 eV, and broad photoluminescence peaks between 350 and 600 nm) NPs. The conversion (SnO_2_ → Sn) reaction temperature was ~450 °C, which is significantly lower than the reaction temperature (1200 °C) of carbothermal Sn production used in industry. The ethanol oxidation products included H_2_, H_2_O, CO, CO_2_, CH_4_, aldehyde, and ethylacetate. On the other hand, uniform nanosized SnO_2_@C core-shells could be prepared by aerobic ethanol oxidation reaction over SnO_2_ NPs. The carbon overlayer was estimated to be ~1 nm on <10 nm size SnO_2_ nanoparticles based on TEM. The core-shell structure showed a good oxygen sensing response and potential applicability to a gas sensor. Under aerobic conditions, the CO oxidation activity of the SnO_2_ NPs showed T_10%_ = 300 °C. This suggests that SnO_2_ NPs have potential applications to an oxidation catalyst.

Overall, the aerobic and anaerobic oxidation reaction could be a versatile method for the fabrication of various nanostructures and provide new insights for understanding the sensing and catalytic reaction mechanism. Uniform nanosized SnO_2_@C core-shells are produced in a much simpler manner and the carbon-support material has very high potential applicability to electrode and sensor materials. The transformation from SnO_2_ to metallic Sn under anaerobic conditions provides a new insights to better understanding the alcohol sensing mechanism. In addition, the unique anaerobic ethanol (alcohol) oxidation reaction could be a promising method for the industrial production of high quality metallic Sn from SnO_2_.

## Additional Information

**How to cite this article**: Kim, W. J. *et al.* Metallic Sn spheres and SnO_2_@C core-shells by anaerobic and aerobic catalytic ethanol and CO oxidation reactions over SnO_2_ nanoparticles. *Sci. Rep.* **5**, 13448; doi: 10.1038/srep13448 (2015).

## Supplementary Material

Supplementary Information
