# Supplementary material for: Metallic Sn spheres and SnO2@C core-shells by anaerobic and aerobic catalytic ethanol and CO oxidation reactions over SnO2 nanoparticles
Source: Sci Rep. 2015 Aug 24;5:13448. doi: 10.1038/srep13448 (PMC4547105; doi:10.1038/srep13448)
Supplement: Supplementary Information [file srep13448-s1.doc]

Supporting Information

**Metallic Sn spheres and SnO_2_@C core-shells by anaerobic and aerobic catalytic ethanol and CO oxidation reactions**

**over SnO_2_ nanoparticles**

Woon Joo Kim,^1^ Sungwoo Lee,^2^ and Youngku Sohn^1,*^

^1^Department of Chemistry, Yeungnam University, Gyeongsan, Gyeongbuk 712-749, Republic of Korea

^2^Center for Research Facilities & Department of Materials Science and Engineering, Chungnam National University, Daejeon 305-764, Republic of Korea

* Corresponding author e-mail:youngkusohn@ynu.ac.kr


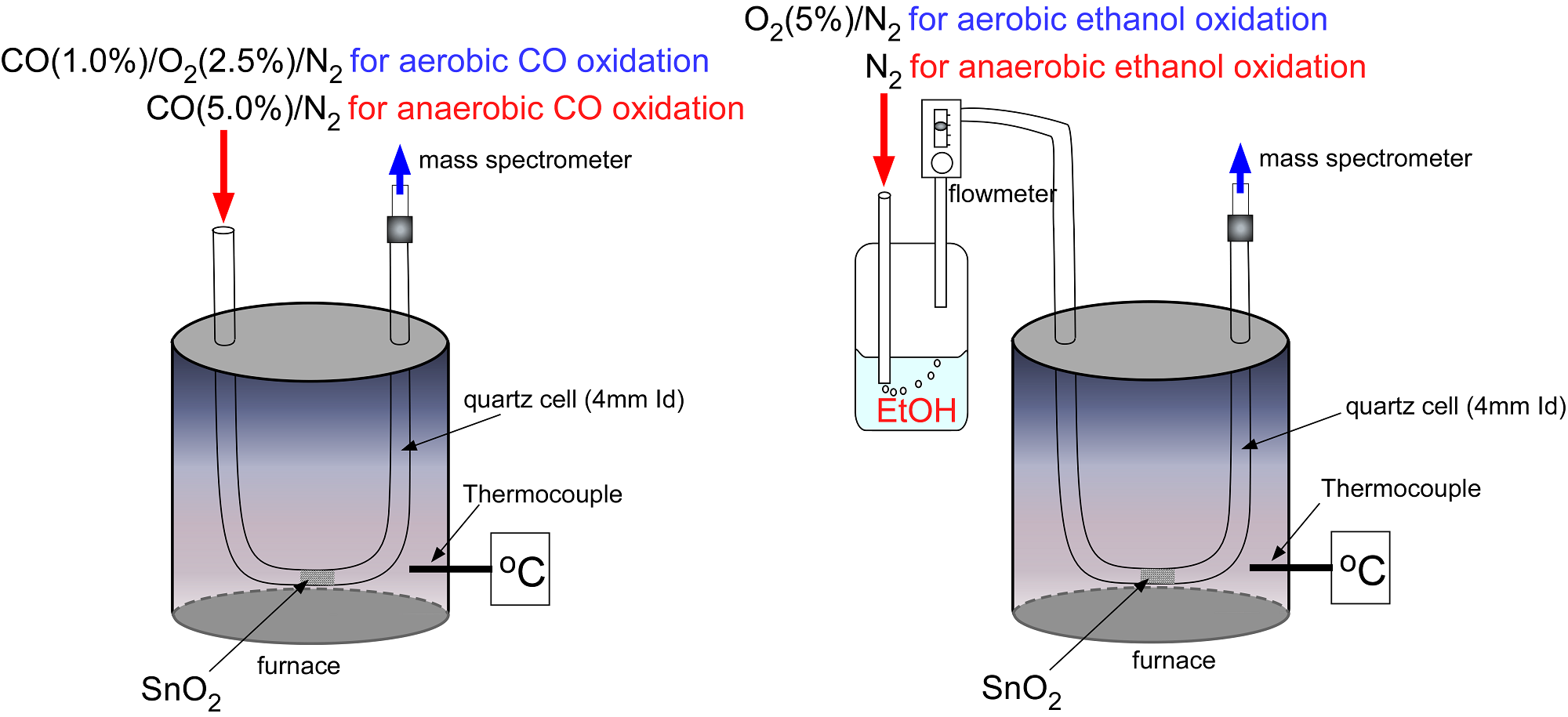


**Figure S1**. Schematics of the experimental setup for the anaerobic and aerobic oxidation reactions.


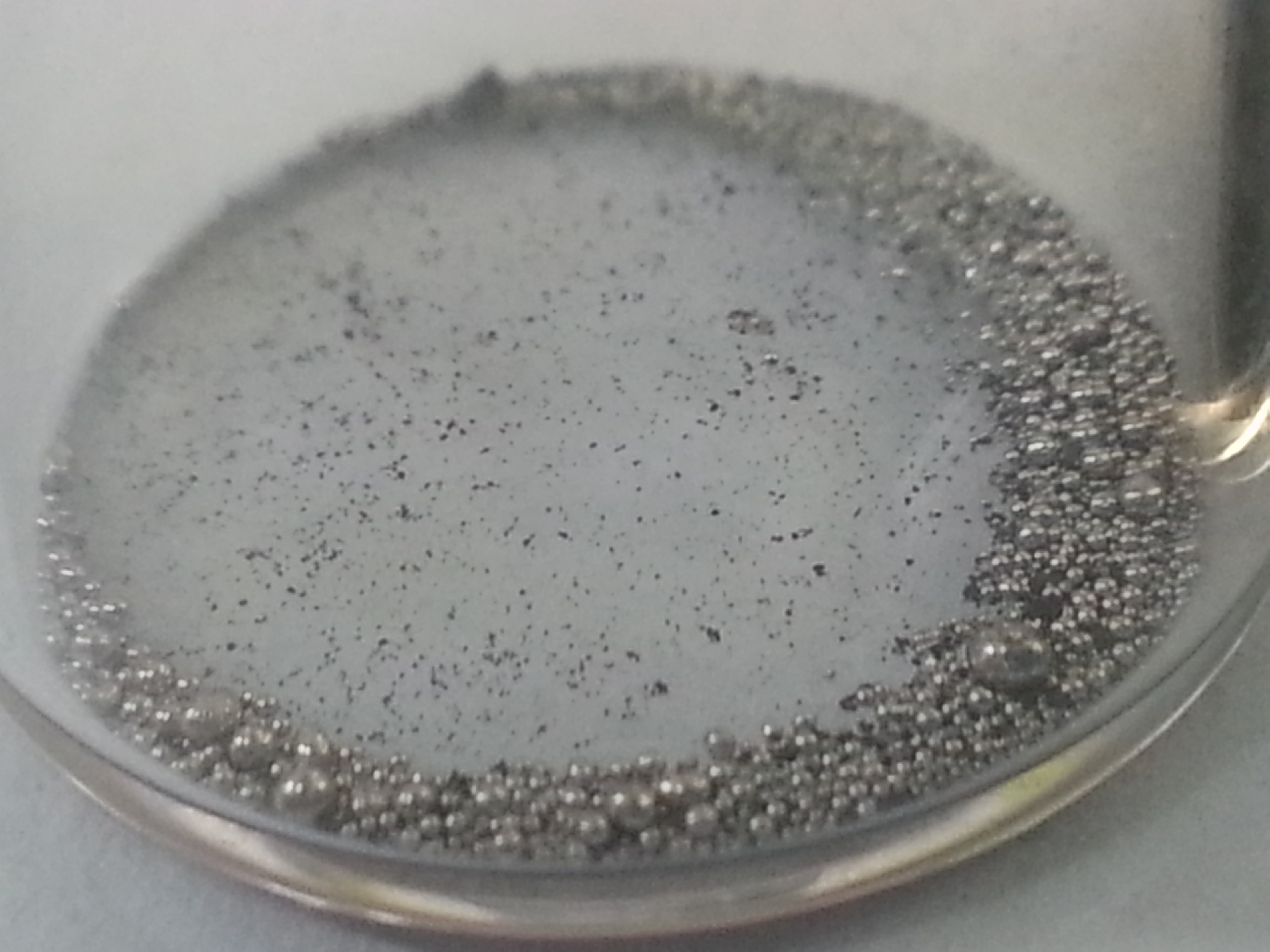


**Figure S2**. Photo of Sn spheres in a vial produced after anaerobic ethanol oxidation reaction over SnO_2_ nanoparticles.


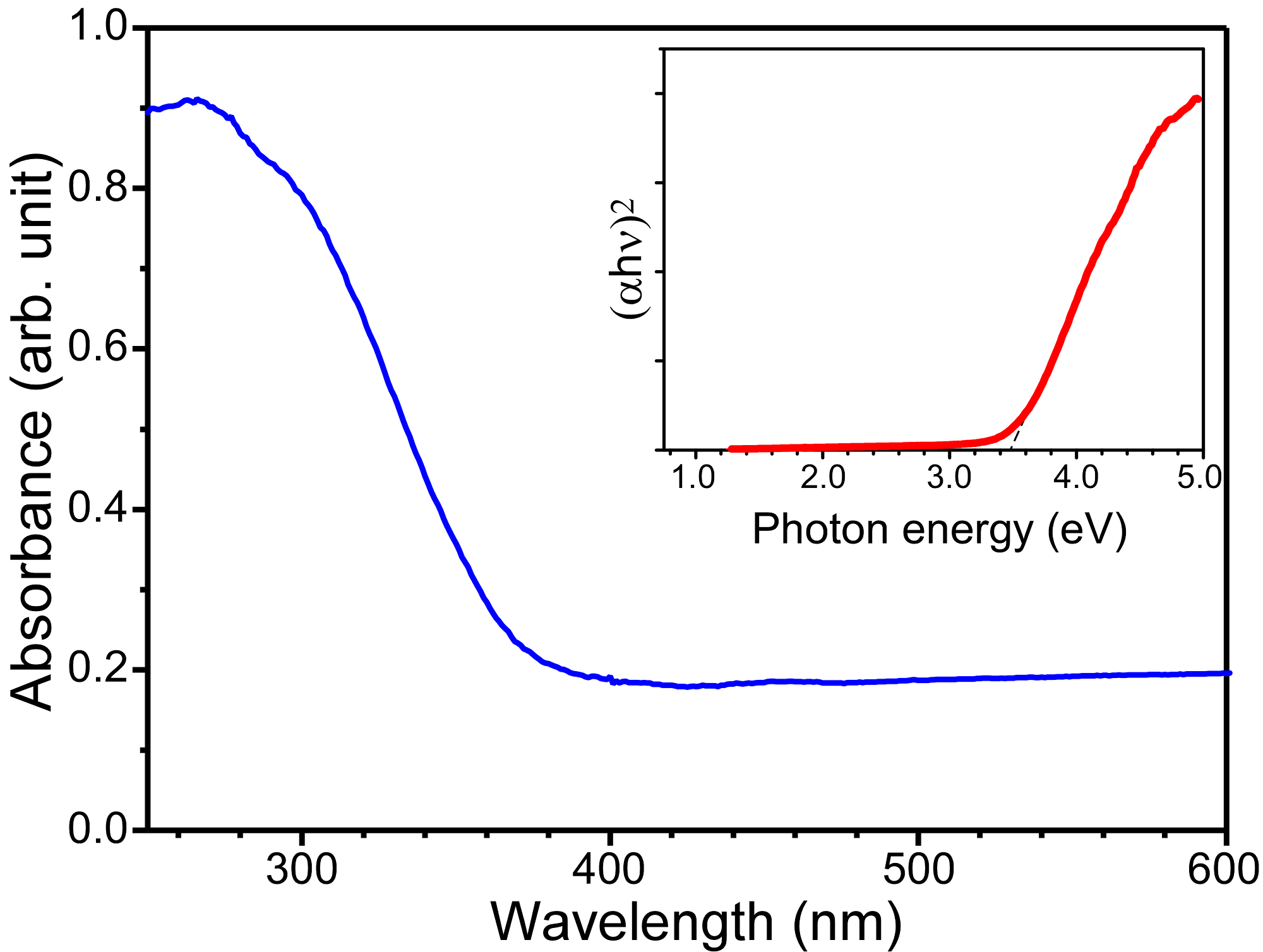


**Figure S3**. UV-Visible absorption spectrum of as-prepared SnO_2_ NPs. The absorbance (Y-axis) was converted from diffuse reflectance data by the Kubelka-Munk method. Inset shows the plot of (αhν)^2^ versus hν.


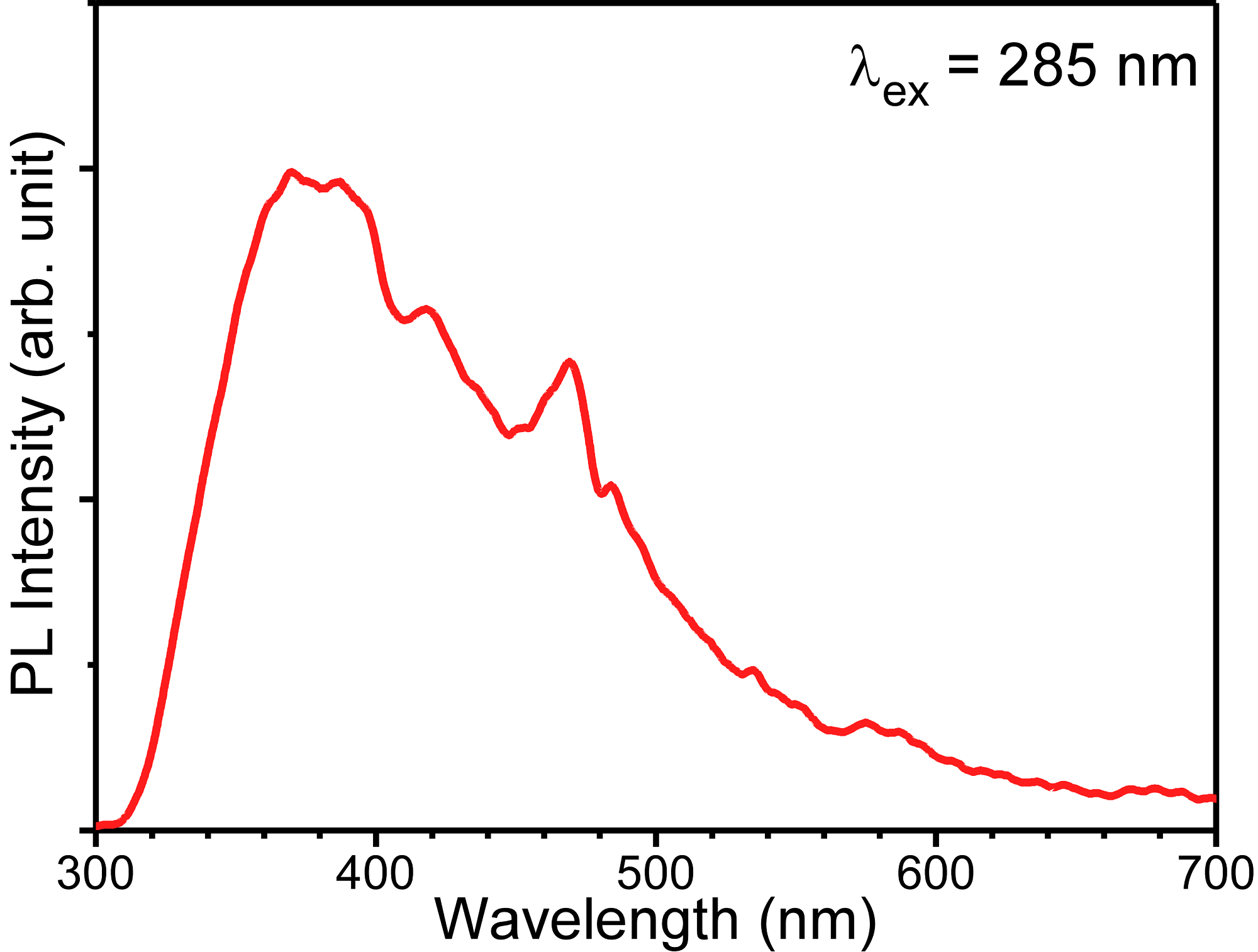


**Figure S4**. Photoluminescence spectrum of as-prepared SnO_2_ nanoparticles at an excitation wavelength of 285 nm.


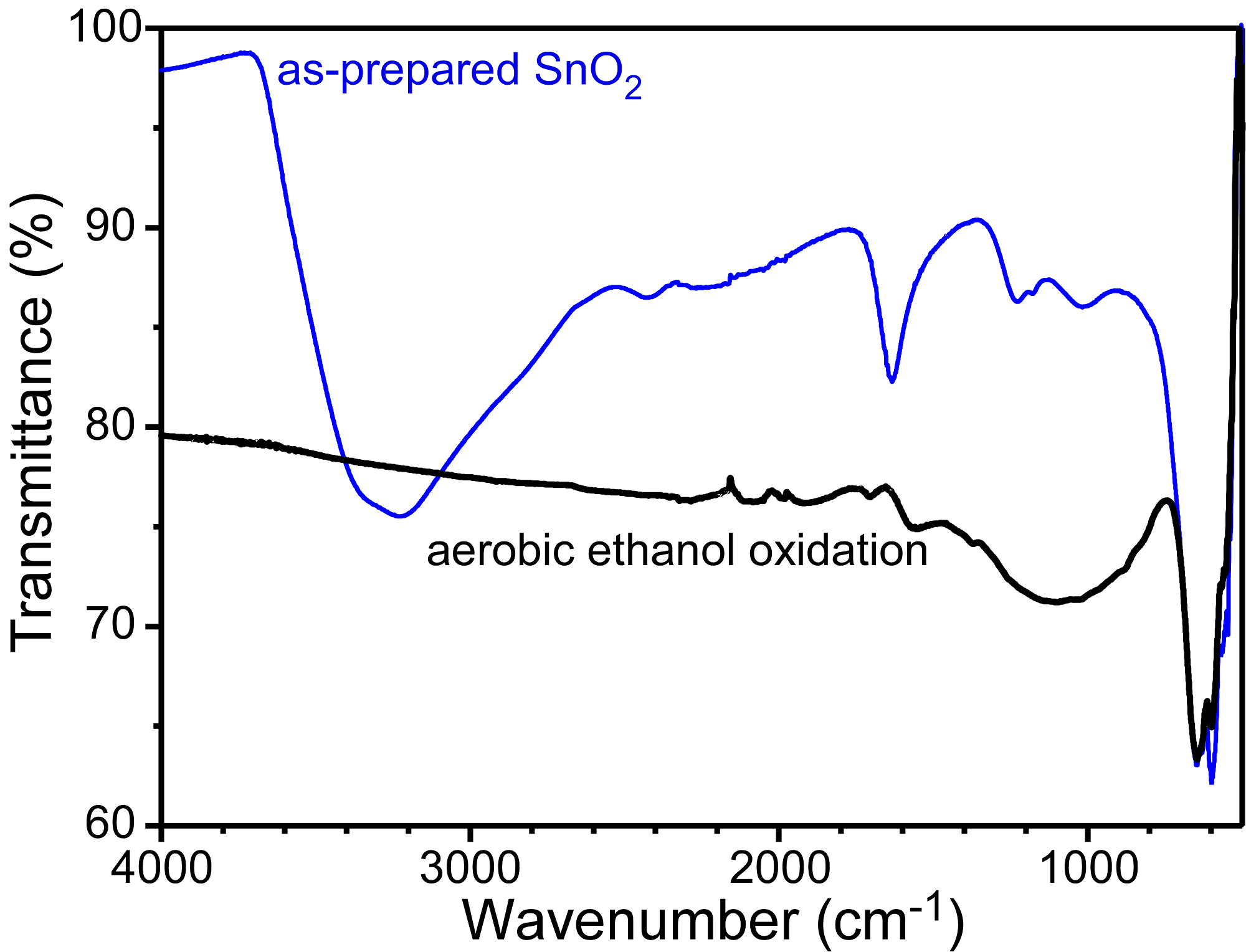


**Figure S5**. FT-IR spectra of SnO_2_ NPs before and after aerobic ethanol oxidation reaction.


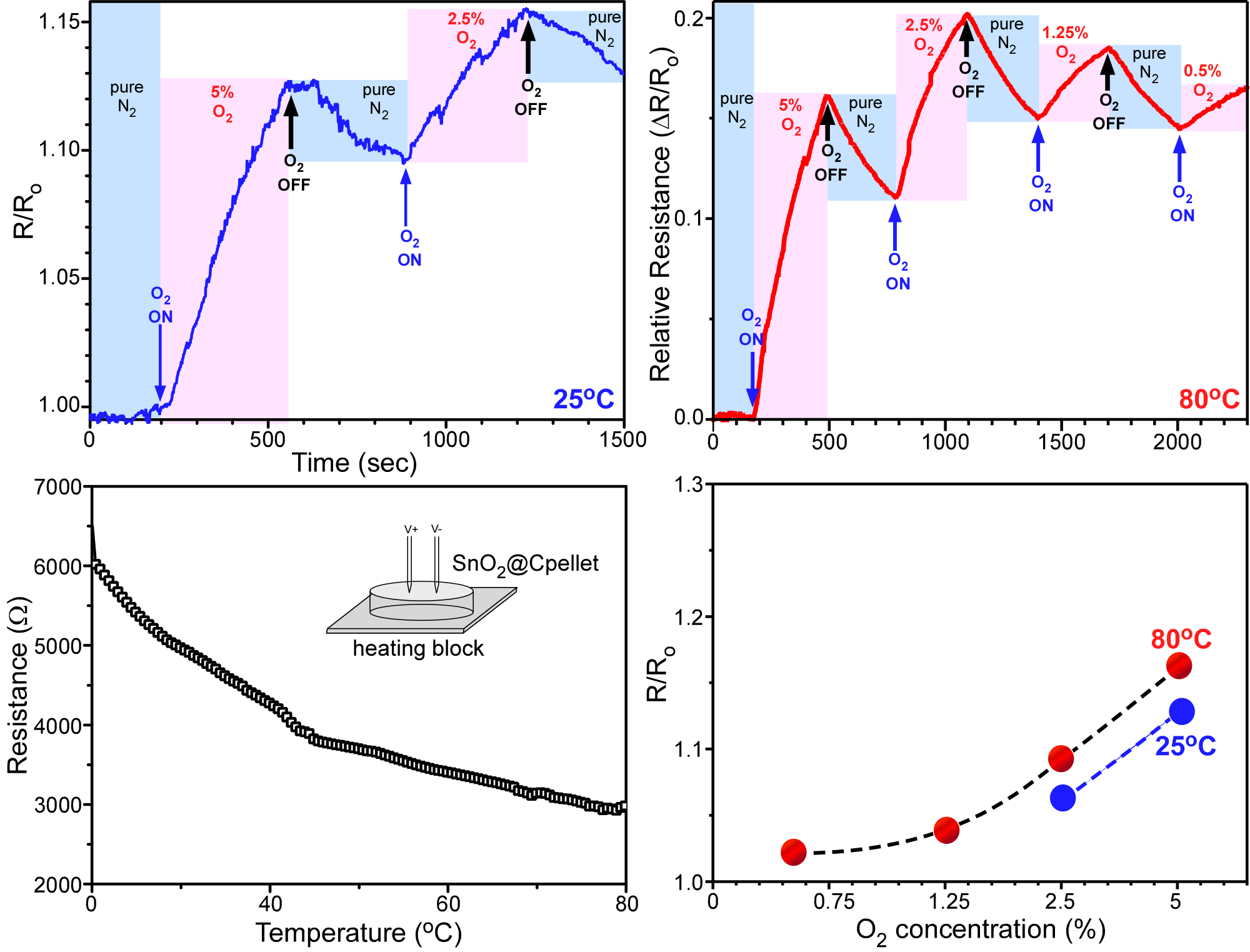


**Figure S6**. Change in surface resistance in response to oxygen concentration at 25 (top left) and 80°C (top right). Surface resistance with temperature (bottom left), and plots (bottom right) of R/R_0_ with oxygen concentration 25 and 80°C


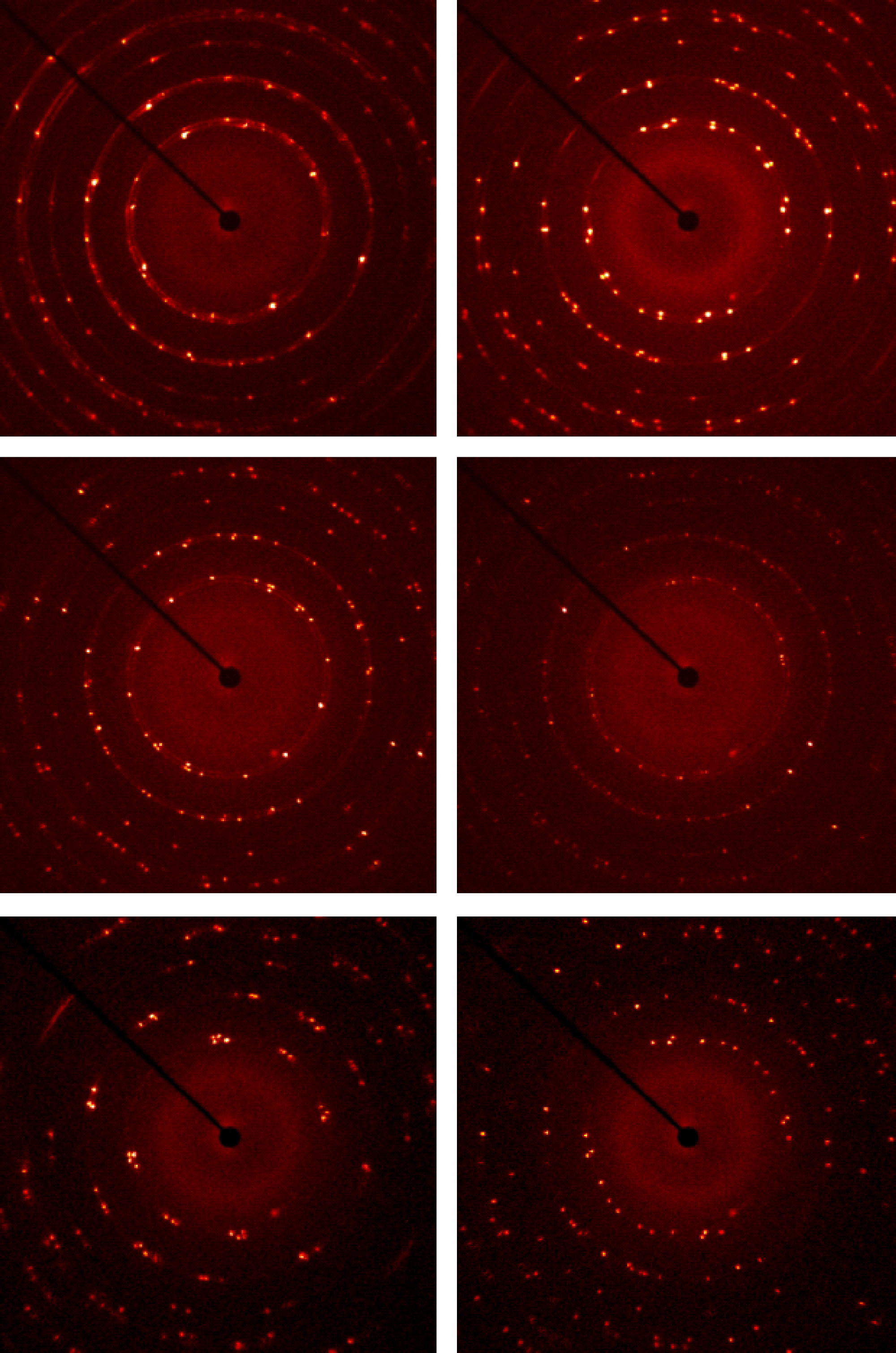


**Figure S7**. Crystal diffraction patterns of Phi 360 image (following 60 second) of various Sn spheres produced after anaerobic ethanol oxidation over SnO_2_ nanoparticles.


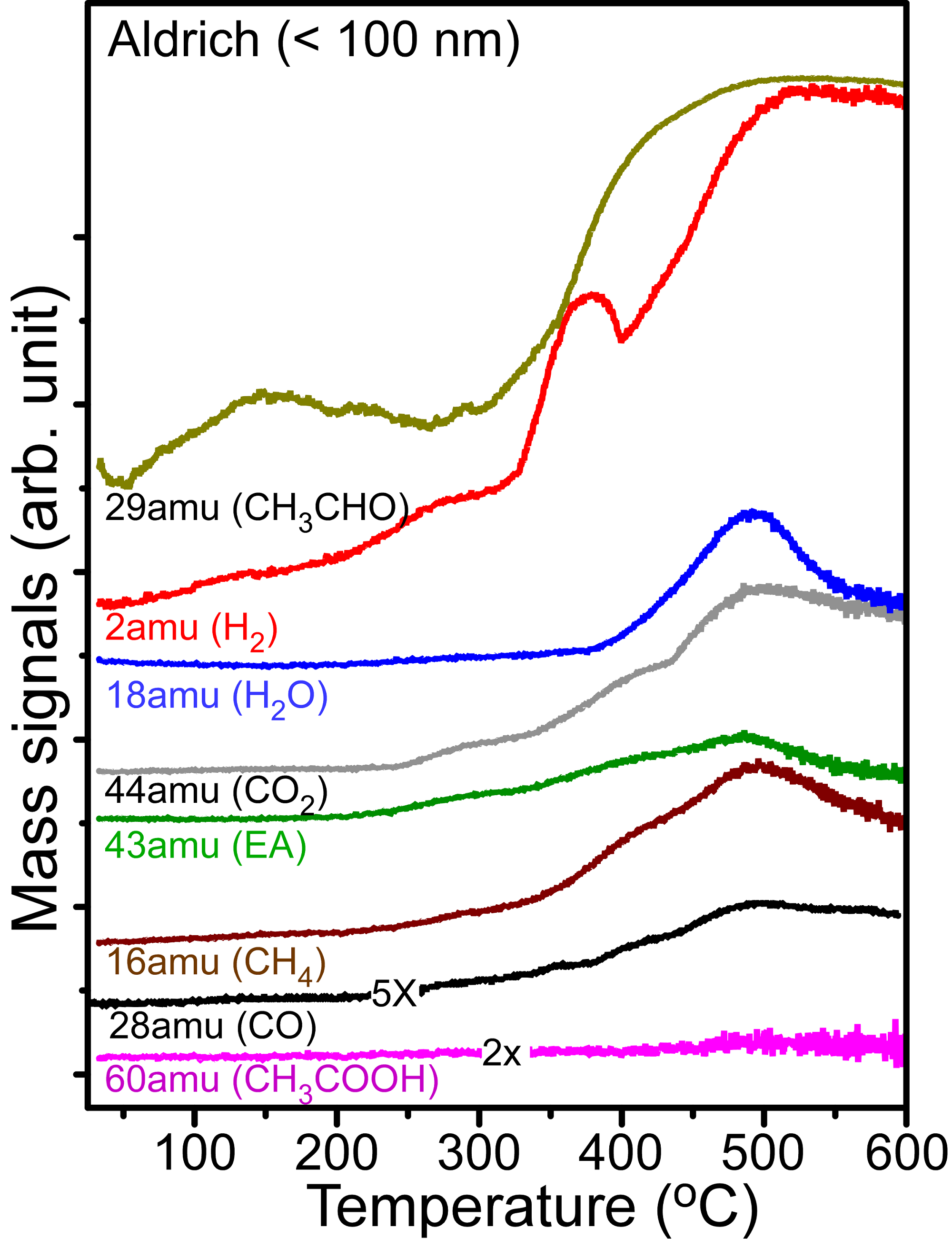


**Figure S8.** Mass profiles of chemical species detected during the anaerobic ethanol oxidation reactions over larger (< 100 nm) SnO_2_ nanoparticles. Metallic Sn spheres SnO_2_@C core-shells were also produced after the reaction.


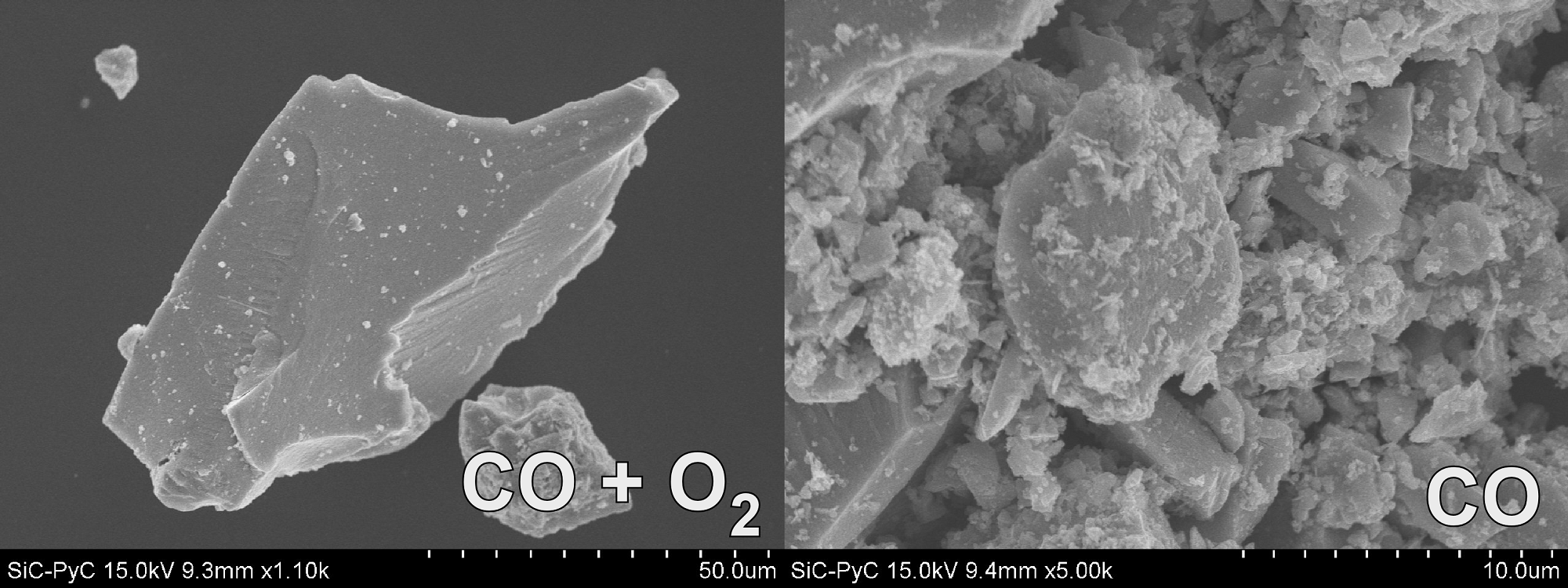


**Figure S9**. SEM images of SnO_2_ NPs after the anaerobic (right) and aerobic (left) CO oxidation reactions. Much bigger particles were formed after the aerobic (left) CO oxidation reaction.


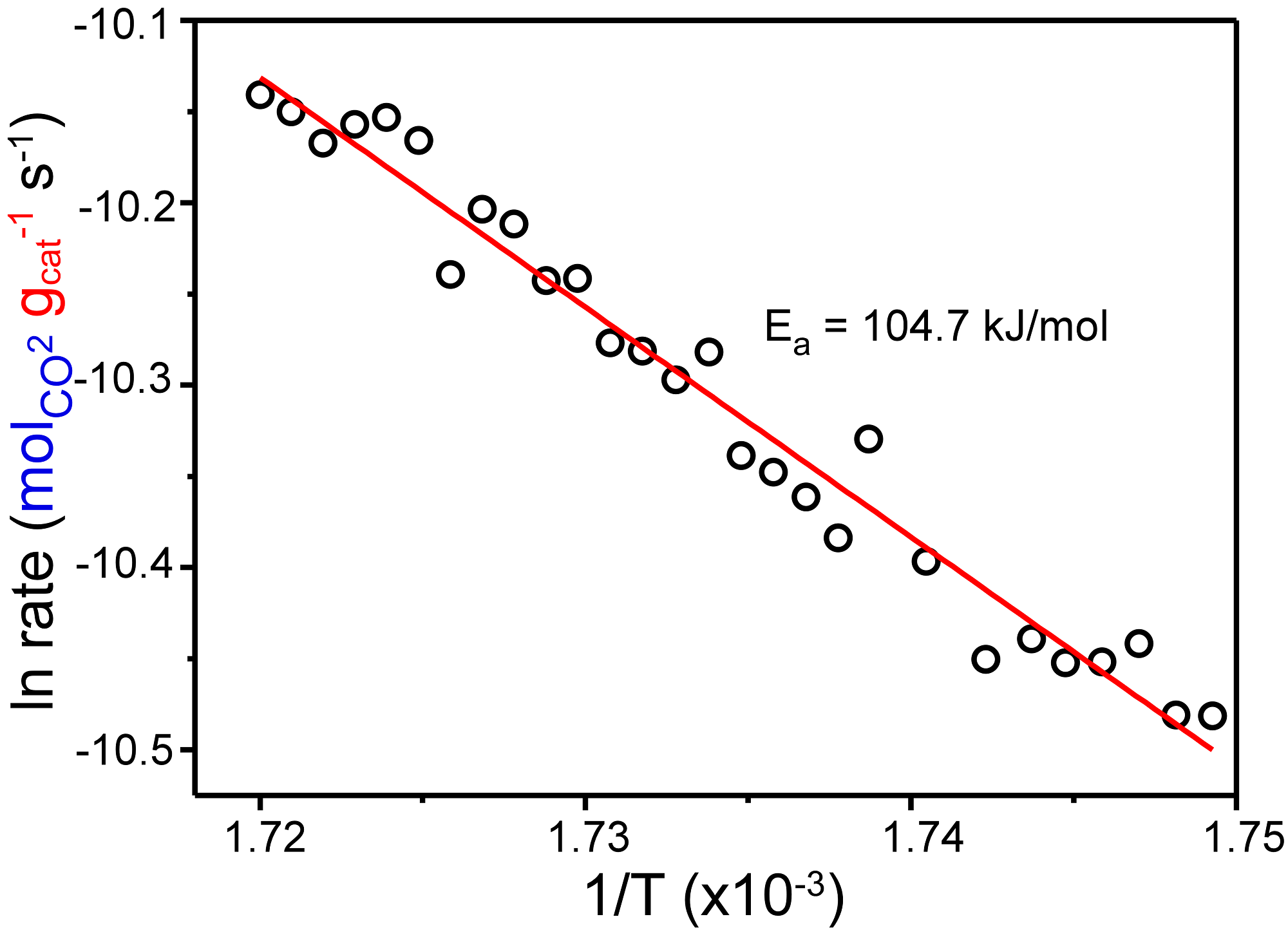


**Figure S10**. Arrhenius plot (ln rate versus 1/T) for the second run aerobic CO oxidation reaction. The activation energy (E_a_) was measured in the CO conversion range of 10–15%.The reaction rate (*v*) was calculated using *v* = (CO flow rate, mol/sec) × (CO conversion fraction)/(weight of the catalyst, g_cat_).


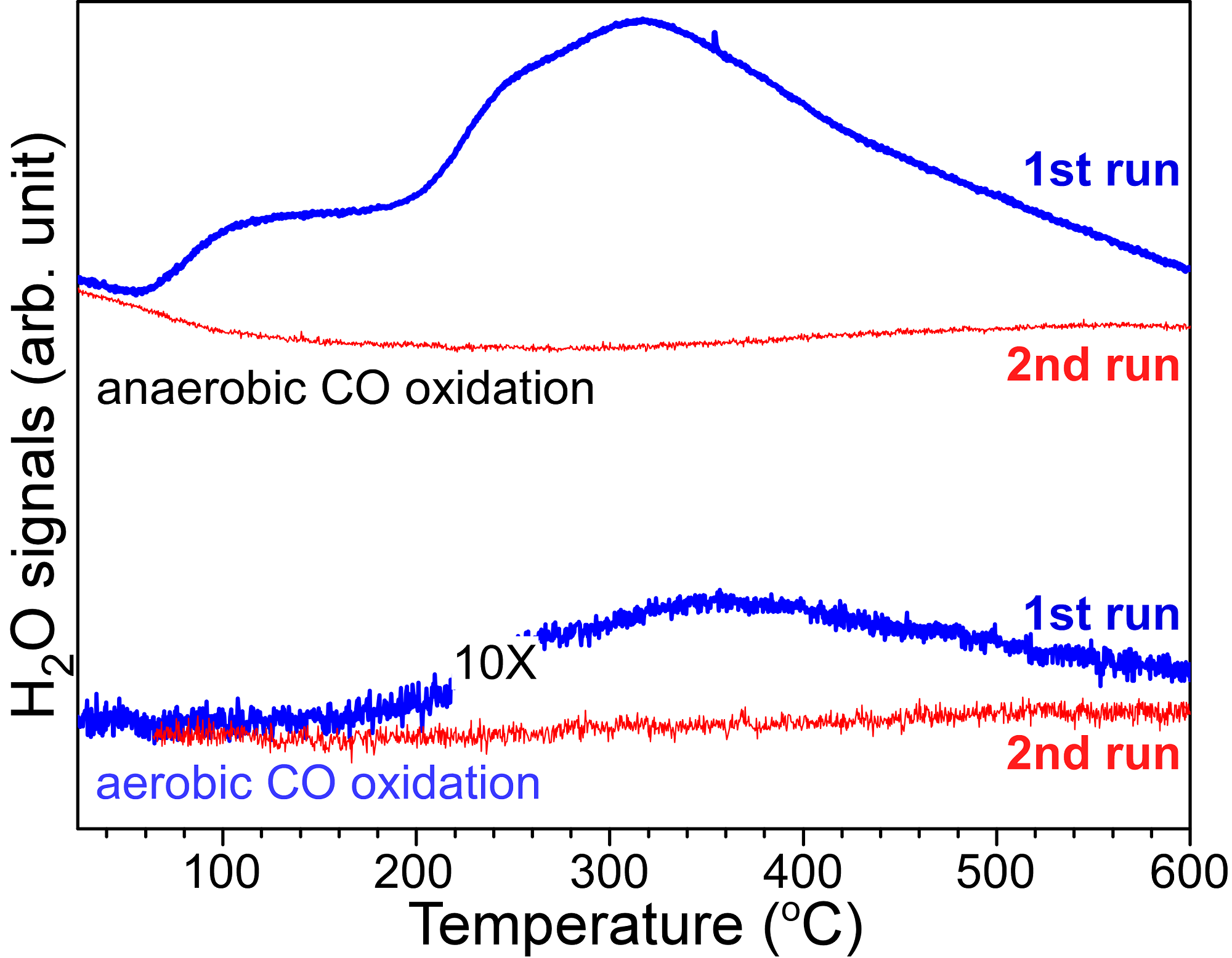


**Figure S11**. Water (mass = 18 amu) signals for the first and second runs of anaerobic (top) and aerobic (bottom) CO oxidation reactions.


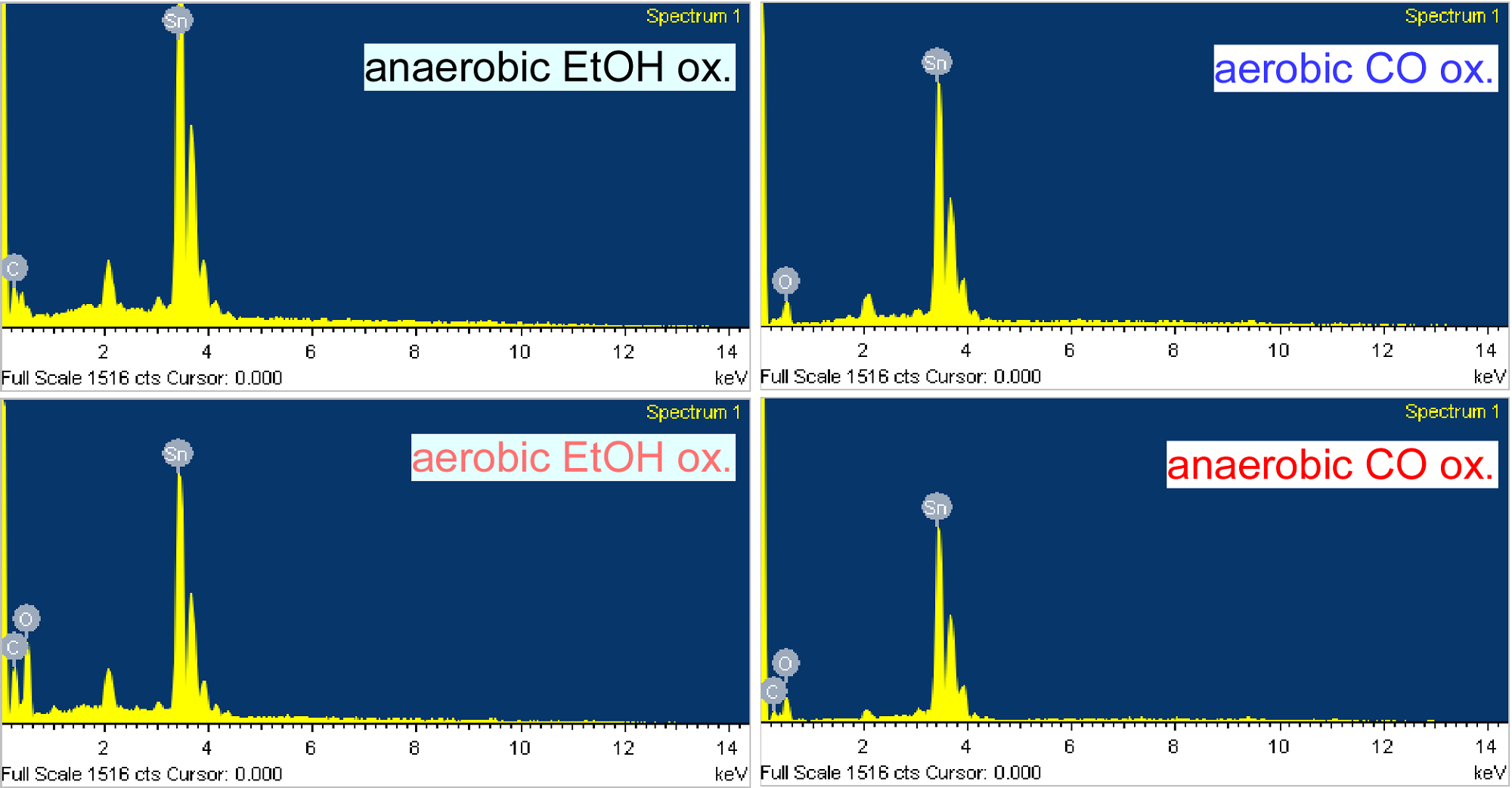


**Figure S12**. Energy dispersive X-ray (EDX) analysis for SnO_2_ NPs after the anaerobic and aerobic CO and ethanol oxidation reactions.
